# Heme oxygenase 1 facilitates cell proliferation via the B-Raf-ERK signaling pathway in melanoma

**DOI:** 10.1186/s12964-018-0313-3

**Published:** 2019-01-11

**Authors:** Liu Liu, Yan Wu, Chunxiang Bian, Muhammad Farrukh Nisar, Mei Wang, Xiangyu Hu, Qingchun Diao, Weiqi Nian, Enwen Wang, Wei Xu, Julia Li Zhong

**Affiliations:** 1 The Base of “111 Project” for Biomechanics and Tissue Repair Engineering, Bioengineering college and Life Science College, Chongqing University, Chongqing, 400044 China; 2 Department of Dermatology, Chongqing First People’s Hospital and Chongqing Traditional Chinese Medicine Hospital, No. 40 Daomenkou St., District Yuzhong, Chongqing, 400011 China; 3 Department of Physiology and Biochemistry, Cholistan University of Veterinary and Animal Sciences (CUVAS), Bahawalpur, 63100 Pakistan; 4 Chongqing Cancer Institute, Chongqing University Cancer Hospital, Chongqing, 400030 China

**Keywords:** HO-1, B-RAF, ERK1/2, Cell cycle, Melanoma

## Abstract

**Background:**

Despite therapeutic advancements (e.g. B-RAF inhibitors) targeting cutaneous melanoma, many cellular processes, including inducible heme oxygenase 1 (HO-1), counteract treatments for malignancies. So there is an urgent need to find biological treatment targets, develop new therapeutic approaches and achieve longer responses. This study aimed to explore the relationship of HO-1 and B-Raf via mediating ERK1/2 signaling on cell cycle in melanoma.

**Methods:**

Immunohistochemistry was applied to evaluate the levels of HO-1 and B-Raf expression in melanoma tissues and adjacent healthy tissues. Co-immunoprecipitation (Co-IP) assessed the interaction of HO-1 with B-Raf. Further study overexpression and knock-down of HO-1 in A375 cell lines, especially knockout HO-1 using CRISPR-Cas9, verified HO-1 regulate cell proliferation in vivo and in vitro. Finally, Western blot analysis and qRT-PCR were performed to investigate the mechanisms by which HO-1 mediates cell cycle by B-RAF-ERK1/2 signaling.

**Results:**

First, histology and Co-IP show that HO-1 interacts with B-Raf directly in melanoma tissue. Further study illustrated that HO-1 overexpression promotes melanoma cell proliferation while HO-1 reduction represses melanoma cell proliferation because of HO-1 affects cell cycle. Mechanistic studies revealed that HO-1 was associated with a marked activation of B-RAF-ERK1/2 signaling and led to CDK2/cyclin E activation, thereby promoting melanoma proliferation.

**Conclusions:**

Our result reveals a previously unknown mechanism that the HO-1-B-RAF-ERK axis plays an important role in melanoma cell proliferation. Therapeutic target on HO-1 could be a novel method for treating melanoma.

**Electronic supplementary material:**

The online version of this article (10.1186/s12964-018-0313-3) contains supplementary material, which is available to authorized users.

## Background

Among various skin cancers, cutaneous melanoma, a malignancy originating from melanocytes, has the highest mortality. Surgery or combinational chemotherapies have been shown to confer only modest survival benefits in advanced melanoma, with a five-year survival rate below 16% in patients with metastasis [1]. Recently, the increased knowledge about the molecular mechanisms of melanoma has revolutionized its treatment. For example, the B-RAF inhibitors vemurafenib and dabrafenib have demonstrated an improvement in both overall survival and progression-free survival [2]. Unfortunately, relapse usually occurs within months after treatment [3]. Thus, there is an urgent need to develop new therapeutic approaches for melanoma treatment and prolong the responses.

Ultraviolet radiation (UVR) is estimated to be responsible for the appearance of approximately 65% of all melanomas [4], while heme oxygenase 1 (HO-1), the inducible form of heme oxygenase, can be induced in response to diverse cellular stress signals such as UVR and reactive oxygen species (ROS) [5]. As an enzyme with antioxidant and antiangiogenic properties, HO-1 is usually expressed at low levels in most tissues under physiological conditions but can be highly induced following exposure to diverse chemical and physical stressors [6]. Furthermore, an increased HO-1 expression level has been reported in different cancers, including brain tumors, chronic myeloid leukemia, melanoma and lymphosarcoma, suggesting a possible contribution of HO-1 to tumorigenesis [7–9]. These data suggest that HO-1 could is a potential biological treatment target. It is known that somatic mutations in B-RAF genes are common in melanoma. Previous studies demonstrated that *B-RAF* is a critical gene in the development of endometriosis [10], and overexpression of wild-type B-Raf is one of the mechanisms underlying the constitutive activation of the MAPK pathway that stimulates the growth of malignant melanoma cells [11]. Moreover, a previous in vitro study showed that HO-1 increasing in a subset of thyroid cancers is associated with tumor aggressiveness and BRAF^V600E^ expression [12].

Cancer is considered a disease involving cell cycle disruption. In normal cells, the cell cycle progression can be constrained, allowing the cells to discontinue cellular division under certain conditions. In contrast, the cell cycle progression is unhindered in cancer cells have unhindered. G1 phase regulation is frequently impaired in cancer cells. Thus, G1-related regulatory proteins are suitable targets for therapy. Similarly, vemurafenib, a small molecule inhibitor of a driver oncogene, binds specifically to the adenosine triphosphate (ATP) pocket of activated BRAF^V600E^, blocks ERK1/2 activation, halts cell cycle progression at the G0/G1 phase and promotes apoptosis. Regulation of cell cycle progression is a complex process and requires the coordinated action of cyclins, cyclin-dependent kinases (CDKs), and CDK inhibitors (CDKIs). Most tumors, including melanoma, have an abnormal G1-to-S-phase transition, mainly due to the deregulated activity of cyclin E, cyclin D, CDK2 and CDK4.

While there is a clear role of activated ERK signaling in inducing cell cycle arrest and supporting cancer cell proliferation, surprisingly little is known about the potential impact of HO-1 on the ERK signaling activity in cancers bearing oncogenic B-RAF. Here, we report a key role of HO-1 in controlling the melanoma cell cycle by regulating B-Raf expression. Endogenous HO-1 and B-Raf are highly expressed in melanoma tissues, and both are colocalized in the cytoplasm of A375 cells. Depletion of HO-1 using small interfering RNA (siRNA) or the CRISPR/Cas9-based blockade of HO-1 activity further inhibited melanoma cell proliferation, both in vitro and in vivo. The cell proliferation induced by promoting HO-1 resulted in ERK1/2 activation. Moreover, blocking HO-1 also induced cell cycle arrest at G0/G1, and overexpression of B-Raf rescued the cell cycle effect of HO-1. Our studies suggest that targeting HO-1 may be therapeutically relevant to melanoma treatments.

## Methods

### Cell culture and treatment

All cells used in the study were cultured in DMEM (HyClone) containing 10% fetal bovine serum and maintained at 37 °C in a humidified 5% CO_2_ incubator. The medium was replaced every 2 days with fresh medium to maintain cell activity. For treatment, cells were seeded in a 60 mm dish. After overnight culture, cells were exposed to UVR (25 kJ/m^2^, 50 kJ/m^2^, 100 kJ/m^2^) or to H_2_O_2_ (40 mM) for 6 h, and the RNA and protein were extracted 4 h or 10 h, respectively, after exposure.

### Tumor samples

Tumor samples were collected from 4 consecutive patients with melanoma who underwent surgical resection at Chongqing Hospital of Traditional Chinese Medicine (Chongqing, China) between August 2016 and August 2017. Informed consent was obtained from the patients. Those patients with preoperative anticancer treatment or with evidence of other malignancies were excluded from the study. The study protocol was approved by the local Ethics Committee of Chongqing Traditional Chinese Medicine Hospital.

### Analysis of cell proliferation

Cell cycle analysis was performed using fluorescence-activated cell sorting (FACS) as previously described. Additionally, clonogenic assays and the CCK-8 assay were conducted as previously described [13].

### Generation of knockout cell lines using the CRISPR-Cas9 technique

Generation of knockout cell lines using the Cas9 technique was performed as previously described [13]. The gDNA for targeting HO-1 with the lentiCRISPRv2 vector was designed as follows: Oligo 1, 5′-CACCGGCTGCTGACCCATGACACCA-3′; Oligo 2, 5′-AAACTGGTGTCATGGGTCAGCAGCC-3′. Protein lysates were then extracted for further analysis.

### Vector construction and lentiviral transduction

The full-length coding regions of 32 kDa HO-1 were amplified by PCR and cloned into the vector pLJM1 (Addgene #19319) for expression. The specific primers were as follows: 5’-ATACCGGTCACGAACGAGCCCAGCACC-3′ (forward) and 5’-GCATGCCTGAATTCACATGGCATAAAGCC-3′ (reverse). To knock down endogenous HO-1, the plasmids were established with pLKO.1 (Addgene #8453) according to the manufacturer’s protocol. The target sequence was as follows: 5’-AATGCTGAGTTCATGAGGAAC-3′ for HO-1. Packaging of these lentiviruses was conducted as previously described [14].

### RNA extraction and qRT-PCR

Total cellular RNA was extracted using TRIzol reagent (TaKaRa) following the manufacturer’s protocol. RNA was reversed transcribed to cDNA using SuperScriptIII (Promega). qRT-PCR was performed with products from Applied Biological Materials Inc. (Richmond, BC, Canada) on a CFX Connect™ Real-Time PCR Detection System (USA). Glyceraldehyde-3-phosphate dehydrogenase (GAPDH) was used as a reference gene. The relative expression levels of mRNA were quantified using the ΔΔCt method.

### Immunofluorescence and immunohistochemistry

Immunofluorescence and immunohistochemistry staining was carried out as previously described [13]. The antibodies were B-Raf antibody (1:100, Abcam), p-ERK antibody (1:200, Cell Signaling Technology), and HO-1 antibody (1:200, Cell Signaling Technology). Slides were observed under a Nikon E600 microscope (Tokyo, Japan) with a digital camera.

### Western blot analysis

Western blotting was performed as follows: the protein lysates were collected from the cells. The tissue lysates were separated on 12% SDS-polyacrylamide gels and transferred onto polyvinylidene fluoride membranes (Bio-Rad). The antibodies used included HO-1, B-Raf, CDK2, cyclin E, phosphorylation of ERK1/2 (p-ERK) and ERK (1:1000) antibodies, while GAPDH (1:2000) was used as a loading control. The Western blot results were further analyzed using a Bio-Rad ChemiDoc™ XRS+ system.

### Xenografted tumor model

All animal experiments were performed in accordance with the ethical guidelines of Laboratory Animal Welfare and Ethics Committee Of the Third Military Medical University. For the analysis of in vivo tumor growth and invasion, 3 × 10^7^ A375 cells in 200 μl of DMEM were subcutaneously injected into each 4- to 6-week-old nude mouse (*n* = 5). A ruler measured the tumor size every 5 days. At 40 days or 45 days after injection, the mice were euthanized, and the tumors were surgically isolated and weighed use electronic load cell scales.

### Statistical analysis

All values are expressed as the mean ± S.D. Statistical analysis of the data was performed by a two-tailed Student’s t-test (*P < 0.05; **P < 0.01).

## Results

### HO-1 interacts with B-Raf directly in melanoma

**Fig. 1:**
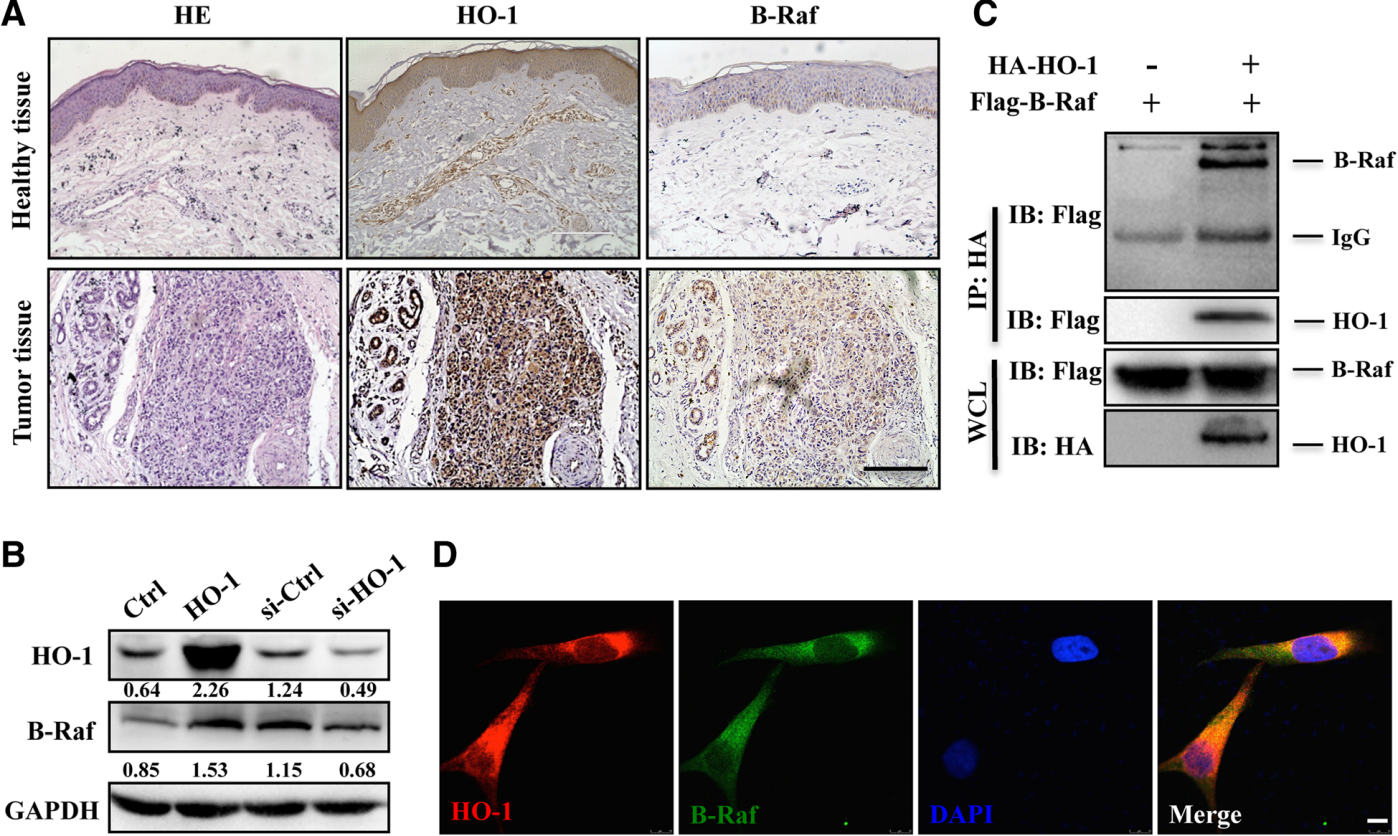
B-Raf is related to HO-1 in melanoma. **a** Hematoxylin and eosin (HE) and immunohistochemical staining of HO-1 and B-Raf were performed on paraffin-embedded sections from adjacent healthy tissues (upper panel) and melanoma tissues (lower panel). Scale bar: 100 μm. **b** The protein levels of B-Raf were measured in A375 cells with HO-1 overexpression or knockdown. **c** The Flag-B-Raf expression plasmid was co-transfected with or without HA-HO-1 into HEK293T cells. HO-1 protein was immunoprecipitated with anti-HA antibody and immunoblotted with antibodies against HA and Flag. **d** Colocalization of B-Raf and HO-1 in A375 cells co-transfected with HO-1 and BRAF expression plasmid. Scale bar: 10 μm

Firstly, we found a higher HO-1 and B-Raf expression in melanoma tissue than that in adjacent healthy tissue by immunohistological analysis (Fig. 1a). In order to explore whether HO-1 and B-Raf have a regulatory relationship in melanoma cells, we assessed the expression of B-Raf in A375 cells by HO-1 overexpression and inhibition. Results showed that overexpression of HO-1 significantly promoted the expression of B-Raf, while inhibition of HO-1 remarkably suppressed B-Raf expression (Fig. 1b and Additional file 1: Figure S1A). Additionally, we found that endogenous HO-1 and B-Raf are colocalized in the cytoplasm of melanoma tissue (Additional file 1: Figure S1B). We then investigated whether HO-1 could interact with B-Raf. HO-1 was transfected alone or together with B-Raf into HEK293T cells for protein-protein interaction analysis by a co-immunoprecipitation assay. As indicated in Fig. 1d & Additional file 1: Figure S1B, HO-1 could be pulled down by B-Raf, and reversely, B-Raf could be pulled down by HO-1. Finally, we confirmed that both exogenous HO-1 and B-Raf are colocalized in the cytoplasm of A375 cells by immunofluorescence staining (Fig. 1f). Taken together, these results suggest that HO-1 interacts with B-Raf directly.

### HO-1 promotes melanoma cell proliferation

**Fig. 2:**
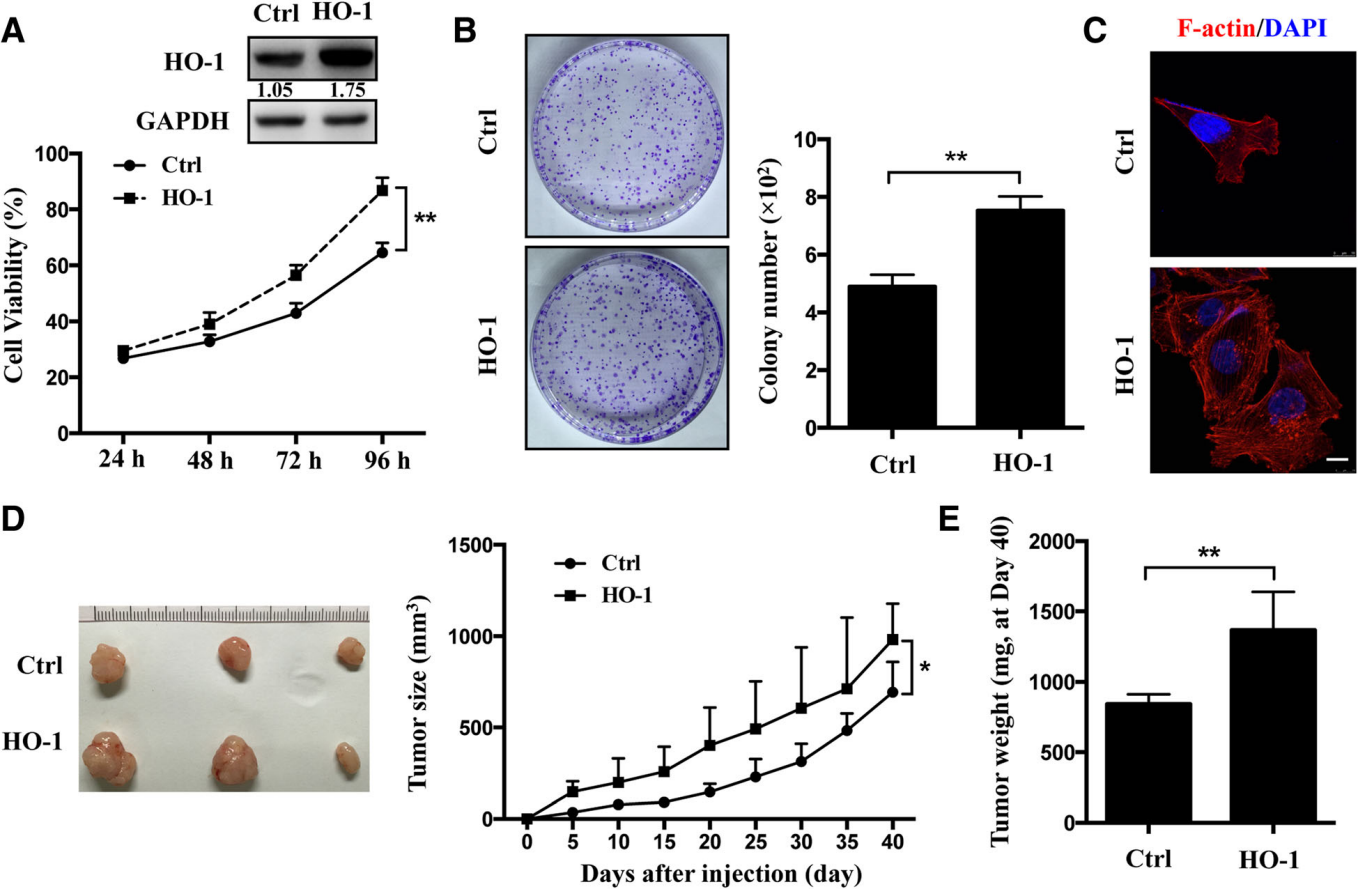
HO-1 promotes cell proliferation in A375 cells. **a** The overexpression efficiency of HO-1 in A375 cells were confirmed by Western blotting. Cell viability was evaluated by the CCK-8 assay. **b** Representative colony formation of A375 cells with HO-1 overexpression. The colony number in each well was determined and statistically analyzed with three independent experiments (*n* = 3). **c** Immunofluorescence staining of F-actin using phalloidin-fluorescein isothiocyanate in scramble and overexpression of HO-1 cells. Scale bar: 10 μm. **d** Representative tumor images were taken from the HO-1 overexpression group and control group. The tumor size (mm^3^) was measured every 5 day for 40 days after injection in both groups. **e** The tumor weight was recorded at the end of the experiment (Day 40) (*n* = 5). *P < 0.05; **P < 0.01 by the *t*-test

It is known that B-Raf can regulate cell growth [15], thus, we hypothesized that HO-1 could modulate cell proliferation by interacting with B-Raf in melanoma. To test this hypothesis, we first established stable A375 cell lines with HO-1 overexpression and then evaluated the cell growth using the CCK-8 assay and a clonogenic survival assay. The overexpression efficiency was confirmed by Western blotting (Fig. 2a). We found that HO-1 overexpression resulted in an accelerated cell proliferation rate (Fig. 2a, b). In addition, we examined the cellular organization of actin filaments using phalloidin fluorescence staining. A more complex array of F-actin in HO-1-overexpressing cells was observed (Fig. 2c). Furthermore, we explored the role of HO-1 in tumorigenesis in vivo. The results show that the average size and weight of tumors significantly increased in HO-1-overexpression cells (Fig. 2d, e). These data show that HO-1 can promote melanoma cell proliferation both in vivo and in vitro.

### Suppression of HO-1 has an anti-proliferative effect in melanoma cells

**Fig. 3:**
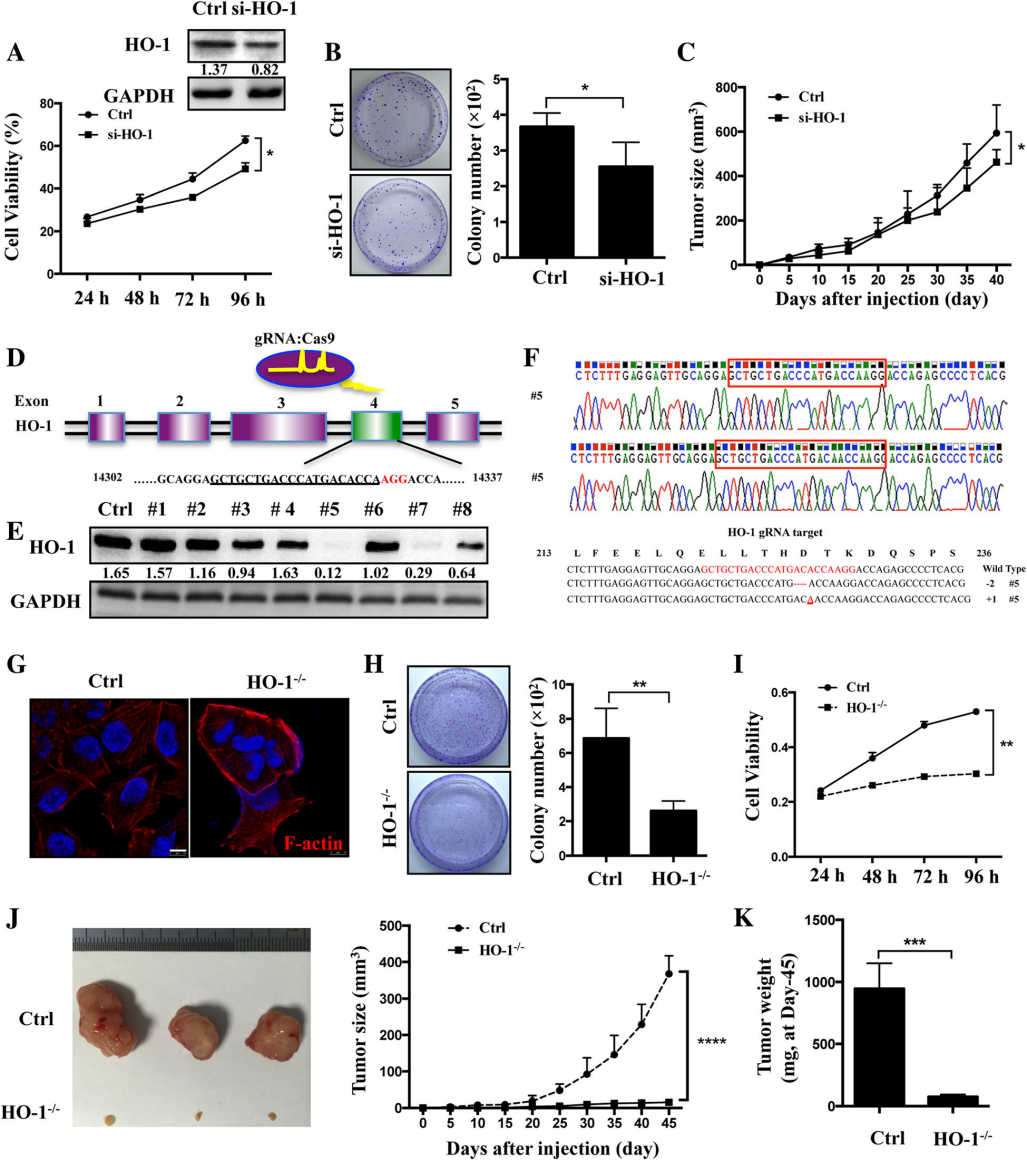
Inhibition of HO-1 represses A375 cell proliferation. **a** The knockdown efficiency of HO-1 in A375 cells was confirmed by Western blotting at 36 h after transfection. Cell viability was evaluated by the CCK-8 assay. **b** Representative colony formation of A375 cells with HO-1 knockdown. The colony number in each well was determined and statistically analyzed with three independent experiments (*n* = 3). **c** The tumor size (mm^3^) was measured every 5 day for 40 days after injection in HO-1 knockdown group and control group. **d** Schematic representation of the genomic DNA structure of the *HO-1* gene. The sequences, which include the AGG PAM targeted by gRNA with the CRISPR-Cas9 system, is shown in red. **e** The HO-1 expression in the knockout cell clone candidates was evaluated by Western blotting. **f** Clone No. 5 had the highest degree of HO-1 reduction and was sequenced, and the sequence was aligned with the wild-type sequence (+: inserted bases; −: deleted bases). **g** Immunofluorescence staining of F-actin using phalloidin-fluorescein isothiocyanate in scramble and HO-1^−/−^ cells. Scale bar: 10 μm. **h** The colony number in each well was determined and statistically analyzed to evaluate the clonogenic survival. **i** CCK-8 assays were used to determine the cell viability of the HO-1^−/−^ cells after treatment. **j** A375 cells with or without CRISPR/Cas9 HO-1 knockout were subcutaneously injected into the flanks of 6-week-old female SCID mice. Representative tumor images were taken. Tumor volumes (mm^3^) were measured every 5 days for 45 days. **k** Tumors were weighed at the endpoint of experiment (Day 45). **P < 0.01; ***P < 0.001 by the *t*-test

Then, we established stable A375 cell lines with HO-1 knockdown and found that silencing of HO-1 decreased cell proliferation rate by both CCK-8 and a clonogenic survival assay (Fig. 3a, b and Additional file 2: Figure S2A). The results in vivo show that the average size and weight of tumors decreased in HO-1-knockdown cells (Fig. 3c and Additional file 2: Figure S2B). Since gene-silencing using the siRNA approach may not sufficient for completely knockout of HO-1, we generated a cell line (A375 HO-1^−/−^) with a homozygous knockout of HO-1 by targeting exon 4 of HO-1 using the CRISPR/Cas9 gene editing system (Fig. 3d). To verify the HO-1 knockout, we performed Western blot analyses and target site sequencing. From 8 clones, we found two homozygous mutant lines and a biallelic 1-nucleotide-inserted and 1-nucleotide-deleted line (#5; Fig. 3e, f) and a biallelic 1-nucleotide-deleted and 9- nucleotide-deleted line (#7; Additional file 2: Figure S2C). Then, we analyzed the cellular morphology of the A375 cells and found spindle-shaped scramble control cells. In contrast, HO-1^−/−^ cells underwent a morphological change into a cobblestone shape with more cell-to-cell contacts (Additional file 2: Figure S2D, E). In addition, we examined the cellular organization of actin filaments using phalloidin fluorescence staining. A more bundled and circumferential arrangement of F-actin in HO-1^−/−^ cells was observed, while the scramble control cells exhibited a more complex array of F-actin (Fig. 3g). The clonogenic survival assay revealed that the clone formation ability was 62.5% lower in HO-1^−/−^ cells than in scramble control cells (Fig. 3h). Moreover, a CCK-8 assay revealed that the HO-1^−/−^ cells grew significantly slower than the control cells (Fig. 3i).

To further evaluate the tumor suppressive ability of HO-1 knockout in vivo, HO-1^−/−^ cells were injected subcutaneously into severe combined immunodeficiency (SCID) mice. The tumor volumes resulting from the HO-1^−/−^ cells were significantly smaller than the tumor volumes resulting from the scramble control cells (Fig. 3j). The tumor growth curves for a five-day period show that the xenograft tumors of HO-1^−/−^cells grew significantly slower than the scramble control cells (Fig. 3j). At the endpoint of the tumor growth experiments at day 45, the tumors from each group were excised and weighed. The HO-1 knockout tumors were much lighter than the control tumors (Fig. 3k). These results suggest that the lack of HO-1 reduces the tumor growth of A375 cells in vivo and *vitro*.

### HO-1 facilitates the progression of the cell cycle through B-Raf

**Fig. 4:**
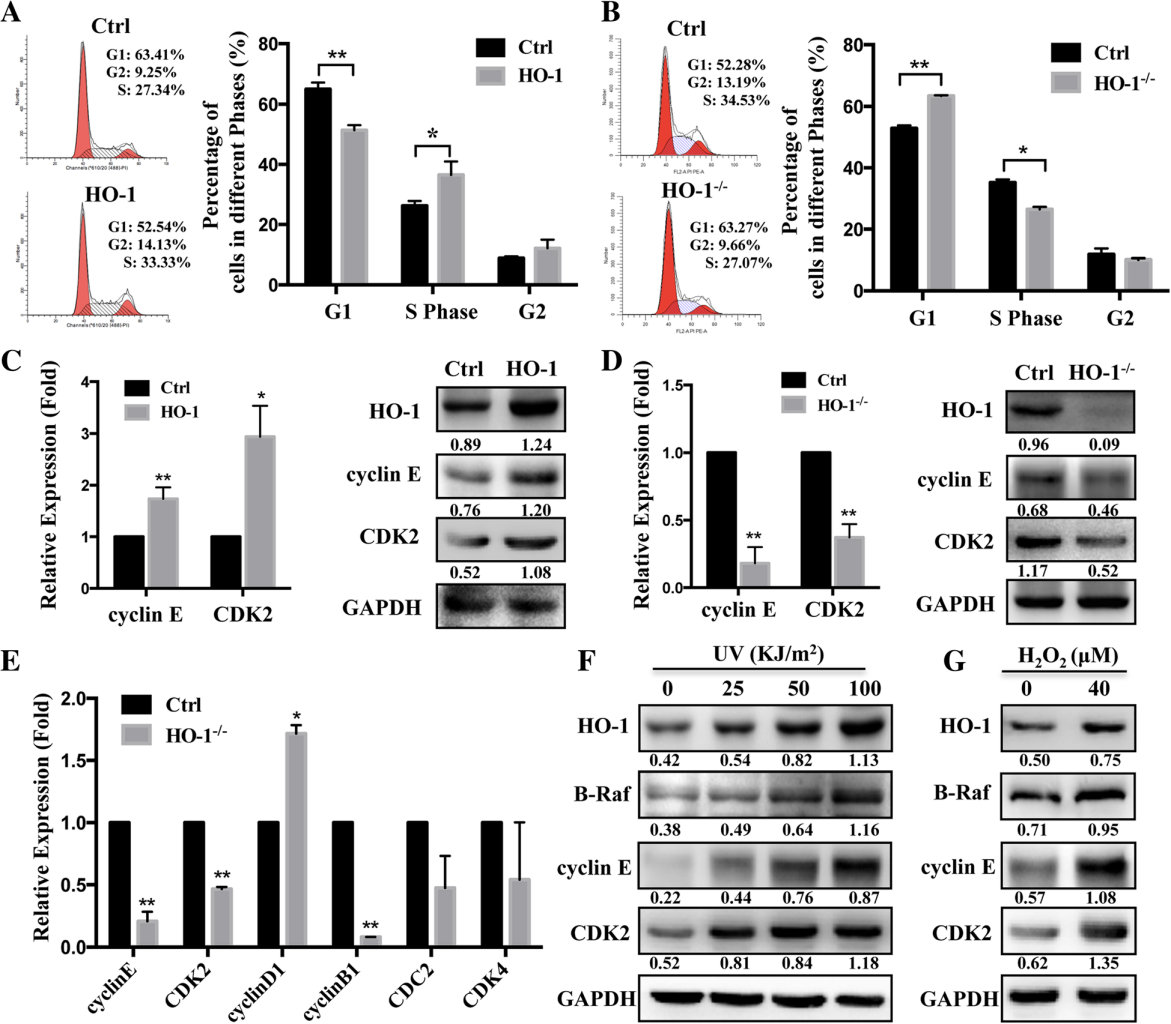
HO-1-induced modulations in cell cycle-related proteins. **a**, **b** HO-1-overexpressing (**a**) and HO-1^−/−^ (**b**) A375 cells underwent cell cycle analysis by flow cytometry (left). Statistical analysis of the cell populations (%) at different stages of the cell cycle (right). **c**, **d** HO-1-overexpressing (**c**) and HO-1^−/−^ (**d**) A375 cells underwent RT-PCR and Western blot analysis of CDK2 and cyclin E. **e** A375 cells with or without CRISPR/Cas9 HO-1 knockout were subcutaneously injected into female SCID mice, q-PCR was performed for cell cycle regulator gene analysis at 45 days after injection. **f** The protein level of HO-1, B-Raf, cyclin E and CDK2 were evaluated by Western blotting in A375 cells treated with UV at 0, 25, 50, 100 KJ/m^2^ after 12 h. **g** The protein level of HO-1, B-Raf, cyclin E and CDK2 were measured in A375 cells treated with 40 μM H_2_O_2_ for 6 h. *P < 0.05; **P < 0.01 by the *t*-test

To further understand the molecular mechanisms linking HO-1 and melanoma cell viability, we assessed cell cycle distributions by flow cytometry. As shown in Fig. 4a, HO-1-overexpression in A375 cells demonstrated a significant decrease in the percentage of cells in the G1 phase and an increase in the percentage of cells in the S phase. Conversely, a decrease in the cell percentage in the S phase and an increase in the percentage in the G1 phase were observed in the HO-1 knockout and HO-1-silenced A375 cells (Fig. 4b and Additional file 3: Figure S3A). A number of key markers, such as CDKs (CDK2, CDK4, CDK2) and cyclin (B, D, E), have been reported to modulate the cell cycle dynamically. We noticed that among these molecules, cyclin E and CDK2 had markedly increased levels after HO-1 overexpression (Fig. 4c and Additional file 3: Figure S3B). As expectedly, the level was decreased after eliminating or silencing of HO-1 (Fig. 4d and Additional file 3: Figure S3C, D). Consistent with these results, the RT-PCR results also showed significant downregulation of cyclin E and CDK2 in the HO-1^−/−^ xenograft tumors (Fig. 4e). It is well known that HO-1 can be induced upon exposure to oxidative stress, such as UVR and H_2_O_2_. We then asked whether the induced HO-1 in response to external stimuli is associated with the cell cycle. We found that, along with HO-1, UV induces B-Raf expression in melanoma cells (A375) as well as the cell cycle markers (Fig. 4f). Similarly, the protein level of B-Raf was also increased after treatment with H_2_O_2_ (Fig. 4g). These results suggest that HO-1 facilitates the progression of the cell cycle by promoting the expression of cyclin E and CDK2.

**Fig. 5:**
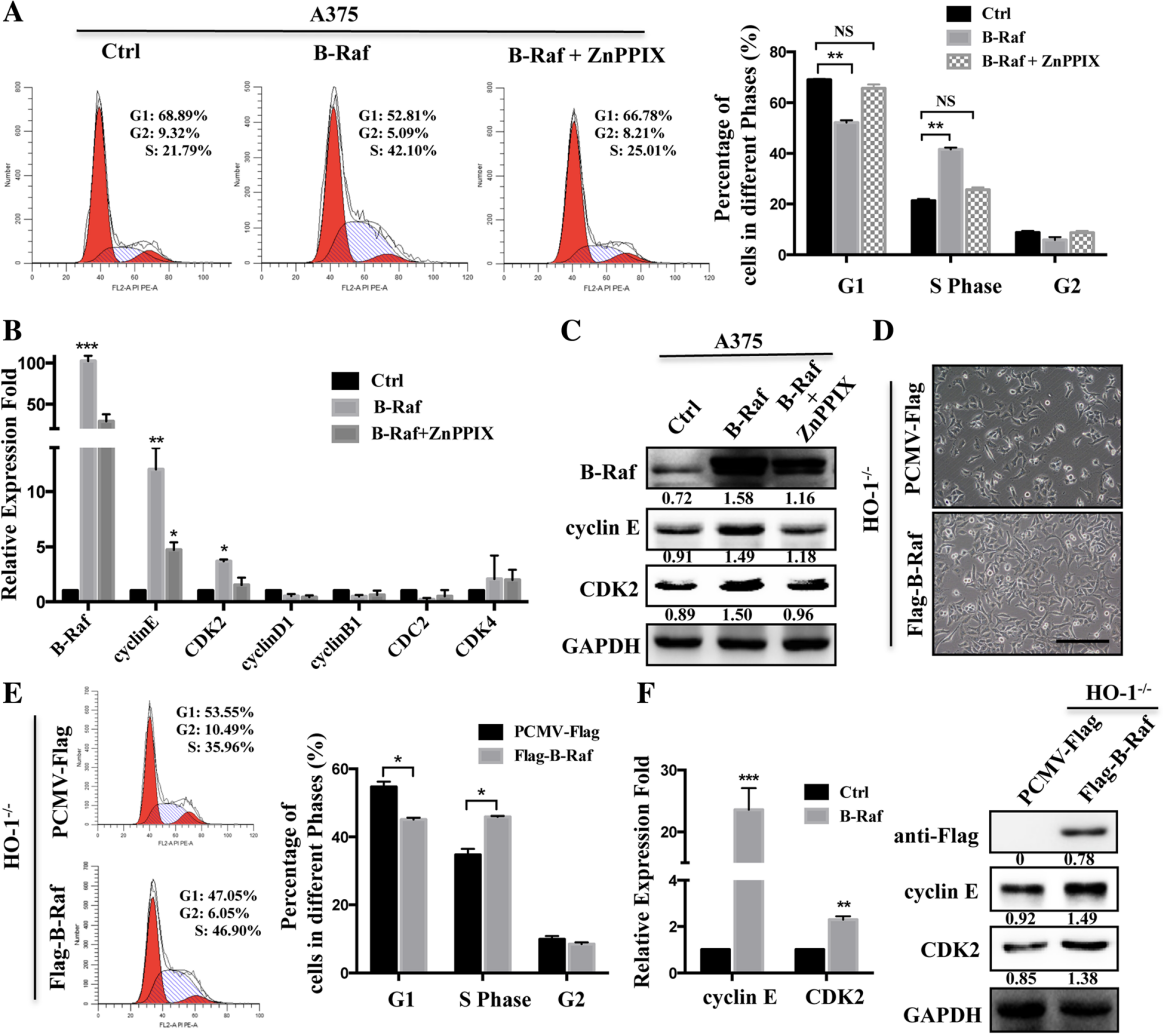
B-Raf reverses the effect of HO-1 knockout on cell cycle. **a**, **b** Flow cytometry analysis result (**a**) and RT-PCR analyses of the indicated cell cycle regulators (**b**) in A375 cells transfected with B-Raf or treated with 20 μM HO-1 inhibitor (ZnPPIX) for 12 h. **c** Protein levels of CDK2 and cyclin E in A375 cells transfected with B-Raf or add ZnPPIX. **d** Comparison of the morphologies of HO-1^−/−^ cells transfected with B-Raf and control vector. Scale bar: 100 μm. **e** Flow cytometry analysis of the cell cycle for HO-1^−/−^ cells transfected with vector and B-Raf. **f** The mRNA level and protein level of CDK2 and cyclin E were measured by RT-PCR and Western blotting. *P < 0.05; **P < 0.01 by the *t*-test

Since we already knew that HO-1 could interact with B-Raf directly, here, we asked whether the HO-1- promote cell cycle through cyclin E and CDK2, is associated with B-Raf. For this purpose, A375 cells were transfected with B-Raf vectors. Cell cycle analysis showed that HO-1 reduction led to substantial accumulation in the G0/G1 phase by A375 cells transfected with B-Raf (Fig. 5a), and this change was accompanied by increases in the mRNA and protein levels of cyclin E and CDK2 (Fig. 5b, c). In addition, the HO-1^−/−^ cells were transfected with B-Raf vectors. B-Raf overexpression not only rescued the HO-1^−/−^ cell morphological changes (Fig. 5d) but also rescued the cell cycle distribution and the related regulator genes (cyclin E and CDK2) (Fig. 5e, f). In summary, HO-1 mediated B-Raf expression and then increased the expression of cyclin E/CDK2, which are involved in regulating the cell cycle and cell proliferation in melanoma.

### Involvement of p-ERK signaling in HO-1-induced proliferation

**Fig. 6:**
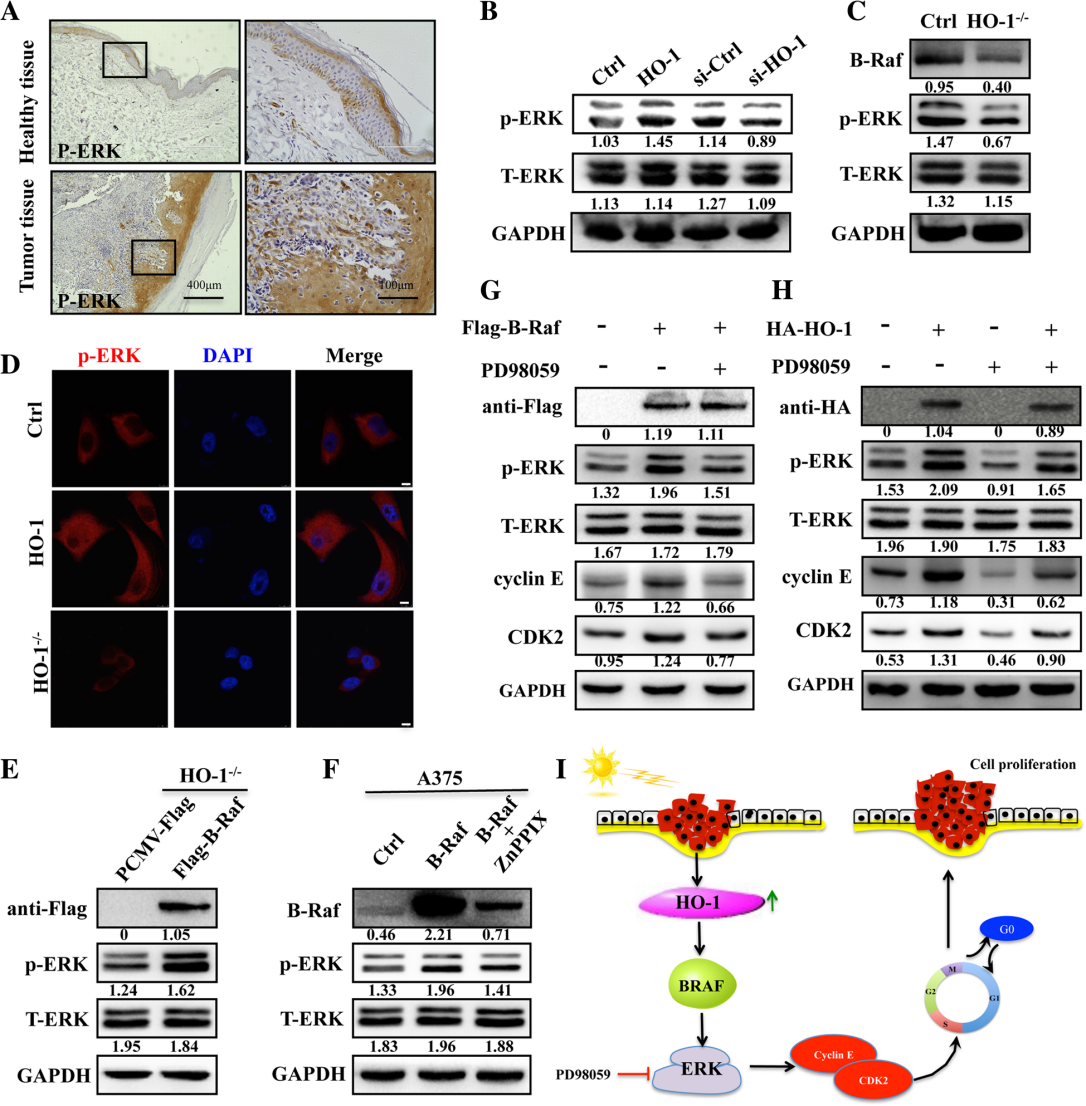
HO-1 regulates the cell cycle through B-Raf-ERK signaling. **a** Immunohistochemical staining for p-ERK in human melanoma tissue. Right panels are magnification from boxed fields in left panels. **b** Protein levels of ERK and p-ERK in HO-1-overexpressing, HO-1-knockdown and (**c**) HO-1^−/−^ A375 cells. **d** Fluorescence analysis of B-Raf expression in HO-1-overexpressing cells and HO-1^−/−^ cells . Scale bar: 10 μm. **e** HO-1^−/−^ cells were transfected with Flag-B-Raf or the PCMV-Flag control as indicated. Forty-eight hours later, the ERK and p-ERK expression was analyzed by Western blotting. **f** Western blot analyses showing that B-Raf can activate p-ERK and that treatment with 20 μM HO-1 inhibitor (ZnPPIX) for 12 h can rescue the p-ERK expression of B-Raf. **g** The cells were pretreated with 20 μM PD98059 (ERK inhibitor) for 18 h after being transfected with B-Raf vector. The lysates were processed to measure p-ERK, ERK, cyclin E and CDK2. **h** HO-1 overexpression in cells treated with 20 μM PD98059 could significantly rescue the cell cycle protein expression changes induced by HO-1. **i** A carton showed HO-1 promotes proliferation by increasing CDK2 and cyclin E expression via B-RAF/ERK signaling

A previous study found that the RAF-ERK pathway is one of the key signal transduction pathways that participate in the cell cycle [16]. The findings raised the question of HO-1 contributes to the cell cycle by regulating p-ERK signaling. To evaluate this possibility, we first analyzed human biopsies using immunohistological staining. The results show dramatically higher p-ERK expression in melanoma tissues than that in the adjacent healthy tissue (Fig. 6a). Furthermore, overexpression of HO-1 resulted in increased p-ERK in A375 cells. In addition, silencing or knockout of HO-1 induced downregulation of p-ERK (Fig. 6b, c). Consistent with these results, immunofluorescence staining also shows upregulation of p-ERK in HO-1-overexpressing cells, while downregulation of p-ERK in HO-1^−/−^ cells (Fig. 6d). In addition, HO-1^−/−^ cells transfected with B-Raf led to greater promotion of p-ERK (Fig. 6e). Interestingly, in A375 cells transfected with B-Raf, the p-ERK and B-Raf levels clearly decreased after treatment with the HO-1 inhibitor ZnPPIX, suggesting that HO-1 is the predominant mediator of MAPK reactivation in the presence of B-Raf (Fig. 6f). To probe the involvement of p-ERK activation by HO-1 in the A375 cell cycle, B-Raf overexpression was performed. Overexpression of B-Raf not only activated ERK signaling but also regulated cyclin E and CDK2 (Fig. 6g). Notably, cyclin E and CDK2, which are required for p-ERK deactivation by the MAPK inhibitor (PD98059), were also suppressed, while HO-1 overexpression rescued the decrease in cell cycle proteins by p-ERK deactivation (Fig. 6h). These results reveal that the HO-1- promote cell proliferation may be mainly mediated via cyclin E/CDK2, through activation of the B-Raf/ERK pathway in melanoma.

## Discussion

Melanomas arise from the pigment-producing cells called melanocytes, which normally reside in the epiderma and extend their dendrites into keratinocytes for melanin transportation and other cell-cell contacts [4]. Melanocytes also interact with skin fibroblasts and basal cells [17]. Thus, many researchers believe that the microenvironment of melanocytes plays an important role in melanoma development [18]. While melanocytes are located in a relatively deep layer of the epidermis, whether UVR causes melanoma has been debated for the past decades [19]. HO-1 is a potent anti-inflammatory intracellular mediator that catalyzes the degradation of heme to iron, biliverdin, and carbon monoxide (CO) [20]. High level of HO-1 expression is observed in many types of cells by stimuli i.e. UVR radiation [21]. Indeed, we found significantly increased HO-1 expression in A375 cells treated with UV. Thus far, numerous data indicate that HO-1 level is high in multiple cancer cells, including sarcoma, melanoma, and pancreatic cancer cell lines [22]. Additionally, the upregulation of HO-1 in melanoma cancer cells is accompanied by the increased production of VEGF associated with cancer development and progression [23]. Similarly, our study showed higher HO-1 expression in skin melanoma tissues. However, studies on the functional role of HO-1 in melanoma are rarely reported, and its function with respect to tumorigenesis and development is poorly characterized. In the current study, we clarified the cause and relationship between HO-1 and B-RAF in terms of the cell proliferation in A375 melanoma cells.

*B-RAF* is a proto-oncogene that is also known as v-raf murine sarcoma viral homolog B1, which belongs to the Raf kinase family [24]. *B-RAF* mutations are associated with numerous types of malignant tumors, where the mutants activate the MAPK signaling pathway constitutively, resulting in uncontrolled cell proliferation and survival [25]. However, other malignant tumors, including primary uveal melanoma and uveal melanoma, demonstrate a lack of *B-RAF* mutations [26]. Some researchers found no mutations in the hotspot regions of *B-RAF* (i.e., exon 15) in primary clear cell ovarian carcinoma [27]. It has been suggested that B-RAF plays an important role in tumor development by binding to specific molecular signaling molecules. Previous studies demonstrated that *B-RAF* is a candidate gene for the development of endometriosis [10], and overexpression of wild-type B-Raf is one of the causes underlying the constitutive activation of the MAPK pathway that stimulates the growth of malignant melanoma cells [11]. Another study performed by our group found that the expression of ^wt^B-Raf was also promoted in melanoma cells (A375, with BRAF^V600E^ mutations) after UV treatment, and the results showed that similar to HO-1, ^wt^B-Raf maintain a high level in skin melanoma tissues. These results led us to propose that HO-1 is related to ^wt^B-Raf in mutant melanoma cells. Our results finally revealed that HO-1 overexpression or knockdown is correlated with the ^wt^B-RAF level in melanoma, and the connection is even more apparent in HO-1^−/−^ cells. Furthermore, the present study found that HO-1 physically interacts with B-RAF and promotes its kinase activity in A375 cells. These results suggest that B-RAF plays a role downstream of HO-1 in promoting cancer development.

It was known that B-RAF contributes to cell growth by regulating the cell cycle [28], and one implication of the observations reported here is that HO-1 may be involved in regulating cell proliferation. Further research by overexpression and knockdown of HO-1 showed that HO-1 promotes cell growth in vivo and in vitro. However, it has been suggested that gene silencing by using a siRNA approach is not sufficient for an in-depth study of the molecular mechanisms of a gene [29, 30]. Furthermore, we used a CRISPR/Cas9 approach to knock out the HO-1 gene in A375 cells, this approach enabled a comprehensive analysis of the role of HO-1 and provided strong evidence that HO-1 exerts promoter effects in A375 cells. Interestingly, the HO-1^−/−^ cells showed a typical morphological phenotype (spindle shaped to cobblestone cell shape change) that was not observed in previous experiments with si-HO-1 where the functional depletion of HO-1 might be insufficient. In addition, the number of A375 cells in S phase of the cell cycle was significantly lower after HO-1 was blocked, while HO-1 overexpression increased this number. Our study revealed molecular mechanisms in which HO-1 induces cell arrest at G0/G1 and showed that genetic depletion of HO-1 exerts antitumor effects. CDKs are protein kinases that regulate the cell cycle by binding to cyclin [31, 32]. The CDK2-cyclin E complex is a major player in cell proliferation that is required for progression through G1 phase and entry into S phase of the cell cycle [33]. Moreover, CDK2 depletion suppresses cell cycle progression in melanoma cells [34]. Here, we noted that knockdown and knockout of HO-1 in melanoma cells significantly downregulated both CDK2 and cyclin E protein levels, whereas HO-1 overexpression could reverse these effects on CDK2 and cyclin E. Finally, via rescue methods in HO-1^−/−^ cells, this study provided proof that the HO^-1-^ cells induced changes in the cell cycle through the cyclin E and CDK2 regulatory proteins were associated with B-Raf expression. The above results indicate that the HO-1-regulated ^wt^B-RAF expression may lead to an increase in the levels of the CDK2-cyclin E complex that stimulates the G1/S transition, which is probably involved in HO-1-induced melanoma cell proliferation.

Some investigators have studied the downstream signaling pathways of B-RAF and found that the RAS/RAF/MAPK pathway is a critical step in the initiation of melanocyte neoplasias [35]. Furthermore, the identification of factors that modulate the balance in favor of ERK may provide additional targets for effective therapy [36]. Our data also confirmed the ERK1/2 was activated in melanoma tissue, as found in previous research [37]. Given that the effects of B-RAF inhibition on cell viability are ERK dependent, we postulate that HO-1-promoted cell growth is mediated through ERK activation. In line with previous studies, a major study suggested the ERK1/2 kinase pathways mediated HO-1 activation and strongly supported the role of HO-1 in directly quenching oxidative stress, normalizing intracellular redox balance, and enhancing cellular survival [38]. Interestingly, HO-1, as an upstream molecule, may promote cell cycle changes via a B-RAF-ERK1/2-dependent mechanism. In addition, suppressing ERK by PD98059 may result in a marked downregulation of CDK2/cyclin E. In contrast, CDK2/cyclin E was restored in the cells transfected with HO-1. This finding may be associated with the activation of B-RAF/ERK signaling, which rescues the G1 cell cycle arrest and promotes cell proliferation by modulating the G1/S transition while enhancing CDK2/cyclin E activities.

## Conclusions

In conclusion, as summarized in Fig. 6i, the present study demonstrates that HO-1 promotes cell proliferation via the B-Raf-ERK signaling pathway in melanoma. HO-1 could be a potential novel anticancer target for melanoma therapy.

## Additional files

Additional file 1: **Figure S1.** B-Raf interacts with HO-1 directly. (A) The mRNA levels of the B-Raf were detected in A375 cells with HO-1 over-expression or knockdown. (B) Colocalization of B-Raf and HO-1 in adjacent healthy tissues and melanoma tissues. The cellular localization of HO-1 (red) and B-Raf (green) was examined by immunofluorescence staining with the corresponding antibodies. Nuclear DNA was stained with DAPI (blue). Scale bar: 100 μm. (C) HA-HO-1 expression plasmid was cotransfected with or without Flag-B-Raf into HEK293T cells. B-Raf protein was immunoprecipitated with anti-Flag antibody, and immunoblotted with antibodies against HA and Flag. The expression of B-Raf and HO-1 in whole-cell lysate (WCL) were confirmed. (TIF 914 kb)

Additional file 2: **Figure S2.** Knockout of HO-1 repressed A375 cell proliferation. (A) Morphological changes in cells with HO-1 knockdown compared to control cells. Scale bar: 100 μm. (B) Representative tumor images were taken from the HO-1 knockdown group and control group. The tumor weight was recorded at the end of the experiment (Day 40) (n = 5). *P<0.05; **P<0.01 by the t-test. (C) Sequencing results for Clone #7. Clone #7 showed the highest degree of HO-1 reduction and was sequenced, and the sequence was aligned with the wild-type sequence (-: deleted bases). (D) Morphological changes in cells with HO-1 knockout compared to control cells. Compared with scramble control cells, cells with HO-1 knockout had more cell-cell contacts. Scale bar: 100 μm. (E) Morphological changes of one cell grown for 4 days for a comparison between the scramble cells and HO-1-/- cells. Scale bar: 50 μm. (TIF 7670 kb)

Additional file 3: **Figure S3.** HO-1 regulated cell cycle-related proteins. (A) A375 cells with HO-1 knockdown cell cycle analysis by flow cytometry (left). Statistical analysis of the cell populations (%) at different stages of the cell cycle (right). All shown values are the mean ± SD of three measurements, which were repeated three times with similar results. (B) HO-1-overexpressing A375 cells underwent RT-PCR analysis for cyclin D1, cyclin B1, CDK4 and CDC2. (C) mRNA levels and protein levels of several cell cycle makers in CDC2 were determined by RT-PCR in scramble control and HO-1-/- cells. *P<0.05; **P<0.01; ***P<0.001 by the t-test. (TIF 914 kb)

## Abbreviations

ATP: Adenosine triphosphate
CCK-8: Cell counting kit
CDKIs: CDK inhibitors
CDKs: Cyclin-dependent kinases
CO: Carbon monoxide
Co-IP: Co-immunoprecipitation
crispr/cas9: Clustered Regularly Interspaced Short Palindromic Repeats (Cas9 nuclease-null)
FACS: Fluorescence-activated cell sorting
H_2_O_2_: Hydrogen peroxide
HO-1: Heme oxygenase 1
HO-1^-/-^: HO-1 knockout cell
MAPK: Mitogen-activated protein kinase
ROS: Reactive oxygen species
SCID: Severe combined immunodeficiency
UVR: Ultraviolet radiation
ZnPPIX: Zn-protoporphyrin-IX
