# Supplementary figures and images for: Heme oxygenase 1 facilitates cell proliferation via the B-Raf-ERK signaling pathway in melanoma
Source: Cell Commun Signal. 2019 Jan 11;17:3. doi: 10.1186/s12964-018-0313-3 (PMC6329143; doi:10.1186/s12964-018-0313-3)

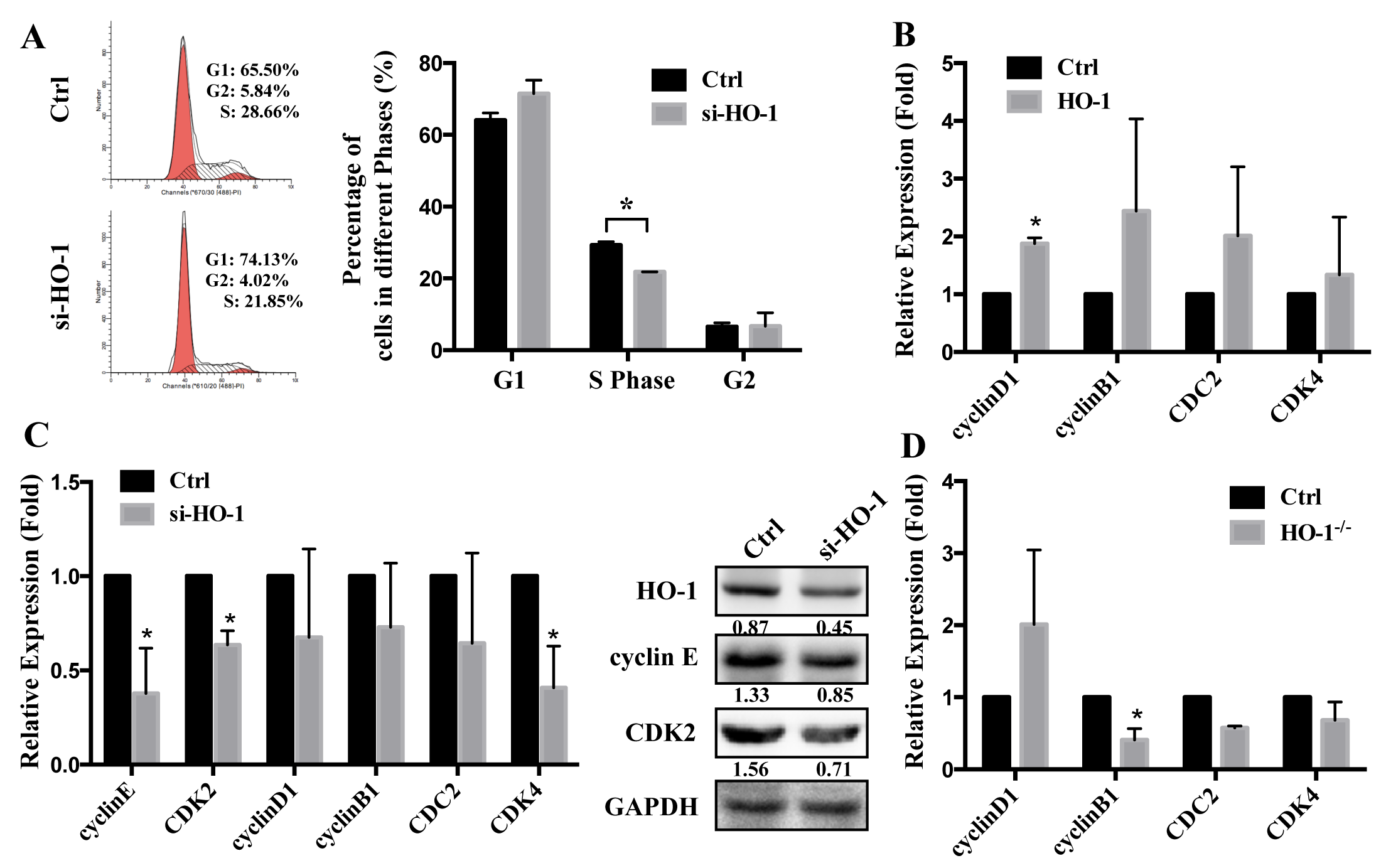

Supplement: Supplementary file 1 — Figure S1. B-Raf interacts with HO-1 directly. (A) The mRNA levels of the B-Raf were detected in A375 cells with HO-1 over-expression or knockdown. (B) Colocalization of B-Raf and HO-1 in adjacent healthy tissues and melanoma tissues. The cellular localization of HO-1 (red) and B-Raf (green) was examined by immunofluorescence staining with the corresponding antibodies. Nuclear DNA was stained with DAPI (blue). Scale bar: 100 μm. (C) HA-HO-1 expression plasmid was cotransfected with or without Flag-B-Raf into HEK293T cells. B-Raf protein was immunoprecipitated with anti-Flag antibody, and immunoblotted with antibodies against HA and Flag. The expression of B-Raf and HO-1 in whole-cell lysate (WCL) were confirmed. (TIF 914 kb) [file 12964_2018_313_MOESM1_ESM.tif]

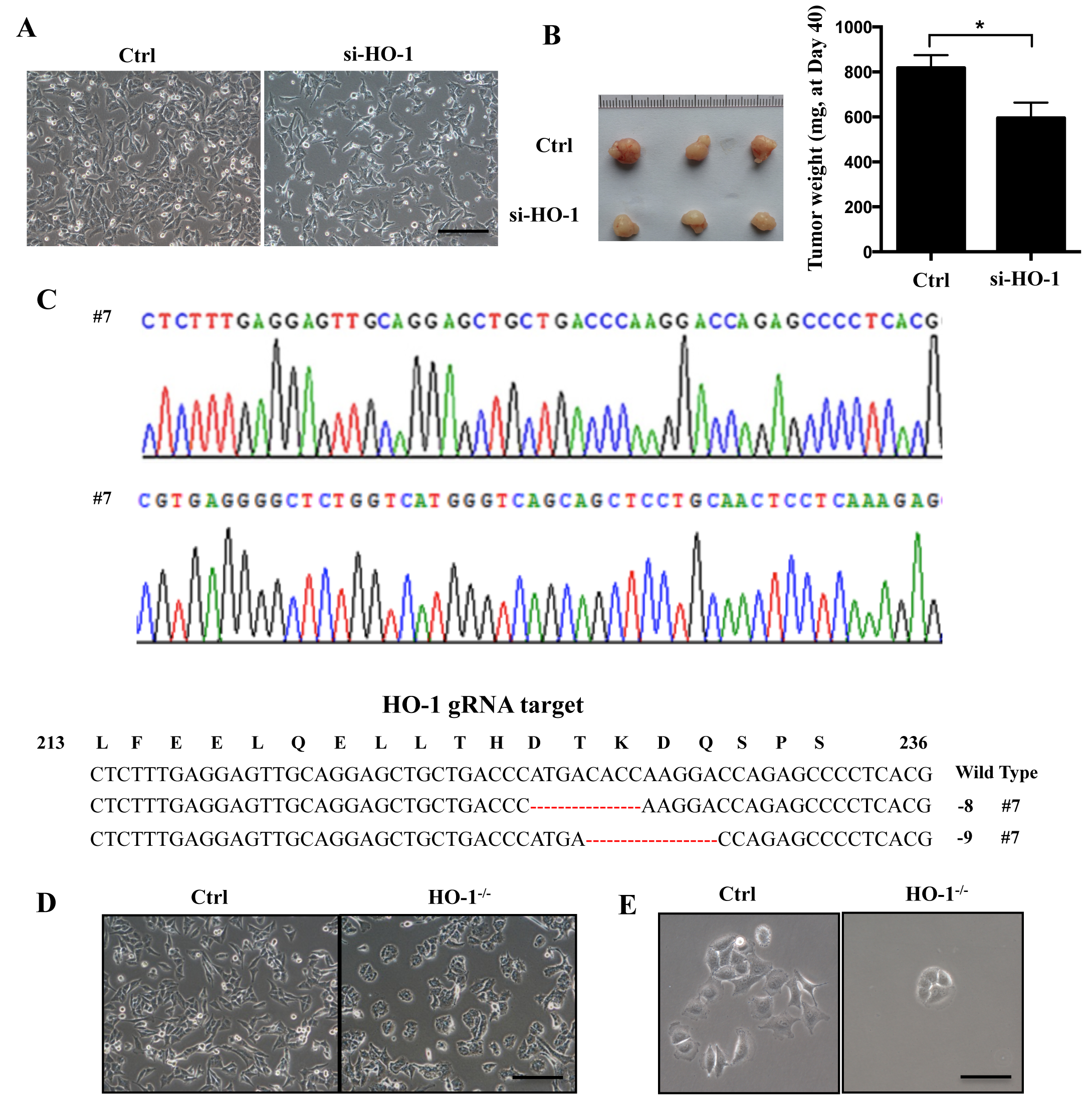

Supplement: Supplementary file 2 — Figure S2. Knockout of HO-1 repressed A375 cell proliferation. (A) Morphological changes in cells with HO-1 knockdown compared to control cells. Scale bar: 100 μm. (B) Representative tumor images were taken from the HO-1 knockdown group and control group. The tumor weight was recorded at the end of the experiment (Day 40) (n = 5). *P<0.05; **P<0.01 by the t-test. (C) Sequencing results for Clone #7. Clone #7 showed the highest degree of HO-1 reduction and was sequenced, and the sequence was aligned with the wild-type sequence (-: deleted bases). (D) Morphological changes in cells with HO-1 knockout compared to control cells. Compared with scramble control cells, cells with HO-1 knockout had more cell-cell contacts. Scale bar: 100 μm. (E) Morphological changes of one cell grown for 4 days for a comparison between the scramble cells and HO-1-/- cells. Scale bar: 50 μm. (TIF 7670 kb) [file 12964_2018_313_MOESM2_ESM.tif]

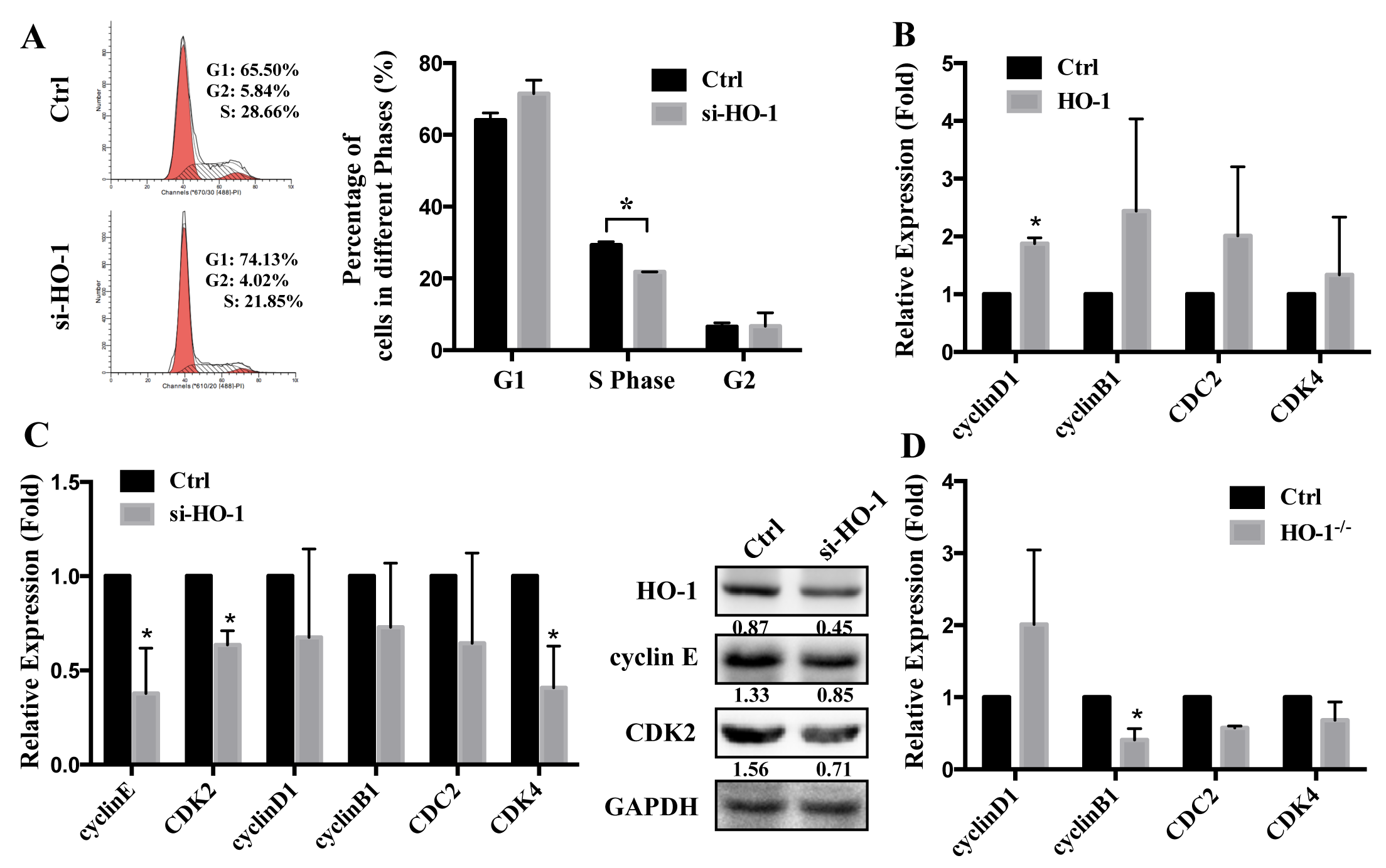

Supplement: Supplementary file 3 — Figure S3. HO-1 regulated cell cycle-related proteins. (A) A375 cells with HO-1 knockdown cell cycle analysis by flow cytometry (left). Statistical analysis of the cell populations (%) at different stages of the cell cycle (right). All shown values are the mean ± SD of three measurements, which were repeated three times with similar results. (B) HO-1-overexpressing A375 cells underwent RT-PCR analysis for cyclin D1, cyclin B1, CDK4 and CDC2. (C) mRNA levels and protein levels of several cell cycle makers in CDC2 were determined by RT-PCR in scramble control and HO-1-/- cells. *P<0.05; **P<0.01; ***P<0.001 by the t-test. (TIF 914 kb) [file 12964_2018_313_MOESM3_ESM.tif]
